# Free Thyroxine Distinguishes Subclinical Hypothyroidism From Other Aging-Related Changes in Those With Isolated Elevated Thyrotropin

**DOI:** 10.3389/fendo.2022.858332

**Published:** 2022-03-04

**Authors:** Enoch J. Abbey, John McGready, Lori J. Sokoll, Eleanor M. Simonsick, Jennifer S. R. Mammen

**Affiliations:** ^1^ Department of Medicine, Johns Hopkins School of Medicine, Baltimore, MD, United States; ^2^ Department of Biostatistics, Johns Hopkins Bloomberg School of Public Health, Baltimore, MD, United States; ^3^ Department of Pathology, Johns Hopkins School of Medicine, Baltimore, MD, United States; ^4^ National Institute of Aging, National Institute of Health (NIH), Baltimore, MD, United States

**Keywords:** subclinical hypothyroidism, hypothalamic-pituitary-thyroid axis, Baltimore longitudinal study of aging (BLSA), free triiodothyronine to thyroxine ratio, older adults

## Abstract

**Background:**

Although a finding of isolated elevated thyrotropin (TSH) often leads to treatment with thyroid hormone, it is not specific to a diagnosis of subclinical hypothyroidism, particularly in older adults. We have previously used longitudinal assessment of TSH and free thyroxine (FT4) to distinguish primary and secondary changes in the hypothalamic-pituitary-thyroid (HPT) axis, an approach which is impractical for clinical diagnosis.

**Objective:**

Identify contemporaneous clinical tests and criteria that predict the longitudinally-derived HPT axis phenotype in those with isolated elevated TSH.

**Methods:**

Using data from Baltimore Longitudinal Study of Aging, participants with over three years of follow up not on thyroid hormone replacement, with a TSH above the reference range and an in-range FT4 at the current visit, and at least 1% per year increase in TSH (mean 6.9% annual increase; n=72), we examined correlations between various clinical factors and the change in FT4 across the phenotypic range from emerging hypothyroidism, with falling FT4, to adaptive stress-response, with rising FT4.

**Results:**

Current FT4 level, but not TSH, Free T3, anti-TPO antibody status, age or sex, was significantly associated with phenotype, determined by the annual rate of change in FT4 in those with elevated and rising TSH, both as a continuous variable (β=0.07 per ng/dL increase in FT4; p<0.001) and in quartiles (p<0.001). We estimated a threshold for FT4 of less than 0.89 ng/dL (11.45 pmol/L; the 24^th^ percentile of the reference range), as predictive of a phenotype in the first quartile, consistent with subclinical hypothyroidism, while a FT3:FT4 ratio below 2.77 predicted a phenotype in the fourth quartile, more consistent with adaptive stress-response.

**Conclusions:**

In those with isolated elevated TSH, a FT4 in the lowest quartile of the reference range differentiates those with developing hypothyroidism from other HPT-axis aging changes.

## Introduction

Thyroid hormone levels are maintained through a non-linear feedback system involving hypothalamic and pituitary regulation of the thyroid gland (constituting the HPT axis). This underlies the rationale for monitoring changes in the pituitary hormone thyrotropin (TSH) as a sensitive marker of early thyroid gland disease. However, the HPT axis also acts to adjust thyroid hormone levels in response to changes in metabolic need that can arise in a variety of conditions. For example, changes in TSH, free thyroxine (FT4), and free tri-iodothyronine (FT3) are found in response to physiologic stressors such as fasting (1) and altered circadian rhythms (2), as well as during episodes of systemic non-thyroidal illness [NTIS (3)]. In addition, our natural history study of the longitudinal trajectories of TSH and FT4 in participants from the Baltimore Longitudinal Study of Aging (BLSA) demonstrated that an elevated TSH could occur with divergent underlying pathophysiology (4). This approach distinguished those with clear evidence of developing hypothyroidism, where a falling FT4 accompanied a rise in TSH, from those in whom the rising and elevated TSH occurred in conjunction with rising FT4. These findings support the conclusion that in older adults, an isolated elevated TSH alone is not sufficient to diagnose subclinical hypothyroidism.

Diagnostic specificity for subclinical hypothyroidism is necessary not only for clinical practice but also in research to determine whether treatment provides benefit. Unrecognized heterogeneity in study populations using TSH-only diagnostic criteria may explain the lack of benefit from thyroid hormone replacement, in particular the failure of a recent large randomized controlled trial to show improvement in symptoms or cognitive function with normalization of TSH in older adults (5). It is unknown whether targeting thyroid hormone use to those with true early primary gland failure would demonstrate benefit from hormone replacement and allow clinicians to adopt a more tailored approach to prescribing levothyroxine supplementation. Unfortunately, longitudinal data, upon which trajectory-based phenotyping relies, is often unavailable in clinical practice. Therefore, we sought to use our well-characterized cohort in the BLSA to investigate whether other markers of thyroid function, measured concurrently with TSH, improve the characterization of HPT axis physiology defined using the combination of longitudinal TSH and FT4 trajectories.

We limited this study to participants with a currently elevated and historically rising TSH, the population in whom a diagnosis of subclinical hypothyroidism is most likely to be considered by a treating physician. Within this subgroup, the HPT axis phenotype can be defined using the range of FT4 trajectories. A decline in FT4 will be found in participants most likely to have true early subclinical hypothyroidism, while a rising FT4 in the setting of a rising TSH is most consistent with a stress-response adaptation. We assessed the ability of concurrent thyroid function tests – TSH, FT4, FT3, and FT3:FT4 ratio – as well as autoimmunity, measured by anti-TPO antibodies, to retrospectively predict the longitudinal trajectory of FT4 in this sub-cohort and therefore to act as a biomarker for underlying HPT physiology.

## Materials And Methods

The BLSA, which began in 1958 as an observational study of normative aging, continues to have a rolling enrollment of healthy volunteers under the auspices of the National Institute on Aging Intramural Research Program (6). Extensive health data is collected for life, on a variable schedule depending on age (every 4 years <60, every 2 years age 60-79, annually from age 80) (7). Study visits typically occur over a three-day stay at the National Institute on Aging Clinical Research Unit in Baltimore, Maryland, beginning in the afternoon on day 1 and are rescheduled for any acute illness. Thyroid function tests are collected in the morning on day 2 of the visit, after an observed overnight fast and prior to morning medication administration.

Testing for TSH [current assay (0.4 – 4.0)IU/mL], FT4 [current assay (0.76 – 1.46)ng/dL], and FT3 [current assay (2.18 – 3.98)pg/mL] was performed for the BLSA in CLIA-certified laboratories. For comparative analyses, laboratory values from before 2011 were normalized to the current assay (Dimension Vista; Siemens Healthineers, Newark, DE) as previously described (4). TPO autoantibodies were measured by the JHH Immunology Core Laboratory using Quanta Lite TPO enzyme-linked immunosorbent assay (San Diego, CA – USA) for the semi-quantitative detection of thyroid peroxidase autoantibodies in participant serum. Negative values range from 0 – 100 units (WHO standard units), with positive values being >100 units (WHO standard units).

As of March 31, 2020, 737 BLSA participants had had at least 3 measures of thyroid function (average 5, range 3-15) while not taking any interfering medications (thyroid hormone preparations, antithyroid medications, oral glucocorticoids, lithium, estrogenic compounds, anti-estrogenic therapies, amiodarone, biotin, and anti-seizure medications, including carbamazepine, phenobarbital, and phenytoin). The current study focuses on those with TSH above the upper limit of the assay-specific reference range and a FT4 within the assay-specific reference range at the most recent visit, as well as a history of rising TSH with an annual rate of change of at least 1% per year. Seventy-two participants met these criteria. Phenotyping to differentiate emerging hypothyroidism versus aging-related changes in this subgroup is defined across the range of change in FT4 from falling to rising trajectories, respectively.

TSH, thyroid hormones, TPO autoantibodies, age, sex, race, smoking history (referred to as smoker if current smoker or quit date < 10 years), walking ability index (a composite walking ability score ranging from 0 to 9, where 0 represents an inability to walk ¼ mile and 9 indicates walking a mile is very easy) and body mass index (BMI), were considered potential predictors in linear regression models with the FT4 trajectory as the outcome. We performed a sensitivity analysis for normalization for the ratio of FT3: FT4 by comparing normalized and non-normalized levels and found no differences in the defined thresholds for diagnosis.

Standard logistic regression was used to estimate the choice of FT4 and FT3: FT4 threshold for distinguishing between the middle (stable) category and the primary gland failure and the middle (stable) category compared to the aging phenotype. Empirically defined thresholds at intervals of 0.01 across the reference ranges for each predictor were tested for statistical significance (FT4, FT3:FT4 ratio). Optimal thresholds were identified using receiver operating characteristic (ROC-statistic).

Ethical approval was obtained from the Institutional Review Boards (IRB) of Johns Hopkins School of Medicine and Intramural Research Program of the National Institutes of Health.

## Results

We identified 72 participants in the BLSA not on thyroid hormone with elevated TSH and reference range FT4 at their most recent visit (the target visit for analysis), in whom the annual rate of increase for TSH was at least 1% per year over at least 3 years of follow up, consistent with progressive TSH elevation. The average TSH at the target visit was 5.4 mIU/L (range 4.05-10.5 mIU/L), and the average annual rate of change in TSH was 6.9% (range 1.2-23.6%). Mean FT4 at the target visit was 0.93 ng/dL (range 0.78-1.24 ng/dL) with annual rate of change from -6.9% to 3%. The mean age in the cohort was 75.6 years, with 51% women and 75% white. Few smoked within the last 10 years (5/72), physical function as measured by walking ability was good, average BMI was 28.6, and 26% had positive TPO autoantibodies (**Table 1**).

**Table 1:** Cohort characteristics across HPT axis aging phenotype quartiles among BLSA participants with isolated elevated TSH.

	Total Cohort(n = 72)	Quartile 1(n = 18)	Quartile 2(n = 18)	Quartile 3(n = 18)	Quartile 4(n = 18)	P-value
**TSH (mIU/L)**	5.4 (1.2)	5.2 (1.0)	5.1 (1.1)	5.7 (1.6)	5.5 (1.1)	0.47
**Free T4 (ng/dL)**	0.93 (0.10)	0.86 (0.05)	0.93 (0.08)	0.92 (0.08)	1.01 (0.13)	<0.01
**Free T3 (pg/mL)**	2.81 (0.30)	2.71 (0.32)	2.92 (0.33)	2.86 (0.26)	2.75 (0.24)	0.15
**FT3:FT4 ratio**	3.04 (0.42)	3.15 (0.41)	3.17 (0.36)	3.11 (0.33)	2.77 (0.45)	0.01
**TPO positivity (%)**	19 (26%)	7 (38.9)	3 (16.7)	3 (16.7)	6 (33.3)	0.30
**Change in TSH**						
** mIU/L/year**	0.2 (0.1)	0.2 (0.2)	0.3 (0.1)	0.2 (0.1)	0.2 (0.1)	0.59
** annual %**	6.9 (5.2)	6.9 (5.5)	8.9 (5.8)	6.4 (5.2)	5.2 (3.4)	0.17
**Change in Free T4**						
** ng/dL/year**	0.00 (0.014)	-0.02 (0.01)	-0.01 (0.002)	0.002 (0.002)	0.01 (0.01)	<0.001
** annual %**	-0.2 (1.4)	-1.9 (1.3)	-0.6 (0.2)	0.3 (0.3)	1.5 (0.7)	<0.01
**Age (years)**	75.6 (12.3)	73.4 (10.6)	74.8 (14.8)	75.5 (12.7)	78.6 (11.1)	0.65
**Female (%)**	37 (51.4%)	8 (44.4)	8 (44.4)	10 (55.6)	11 (61.1)	0.68
**White (%)**	54 (75%)	14 (77.8)	15 (83.3)	15 (83.3)	10 (55.6)	0.17
**BMI (kg/m^2^)**	28.6 (4.3)	28.8 (3.8)	27.8 (3.0)	29.0 (5.4)	28.8 (4.8)	0.87
**Smoker (%)**	5 (6.9%)	2 (11.1)	0 (0)	3 (16.7)	0 (0)	0.12
**Walking Index** (0–9)	8.0 (2.0)	8.4 (1.5)	7.8 (2.5)	8.5 (1.3)	7.2 (2.4)	0.33

Results are given as mean (SD) or percentages (%).

TPO positivity, the presence of anti-thyro-peroxidase autoantibodies; BMI, body mass index; Smoker, current or within the past 10 years; Walking index is a composite with a higher score meaning better physical function.

Longitudinal trajectory analysis to determine the HPT-axis aging phenotype in this cohort of participants with a history of rising TSH varies by the change in FT4. A declining FT4 and rising TSH is consistent with subclinical hypothyroidism, while a rising FT4 with rising TSH suggests a form of stress response or aging adaptation. We, therefore, analyzed the relationship between thyroid function tests, TPO status, and other potential covariates such as age and sex with change in FT4 as a continuous variable. In regression analysis with TSH, FT4, FT3, and TPO antibody status, only FT4 was associated with the change in FT4, and thus with underlying physiologic phenotype (β=0.07 (0.04, 0.10), p<0.001; **Table 2**). The ratio of FT3:FT4 was negatively associated with the change in phenotype (β=-0.012 (-0.020, -0.004), p<0.01), driven by the FT4 association. None of the analyzed potential covariates, including age, sex, race, BMI, smoking, or physical function, were associated with the change in FT4. The relationship between phenotype and FT4 remained positive and statistically significant with both partial adjustment for other thyroid variables and full adjustment, including all clinical factors.

**Table 2:** Association of current clinical parameters with Free T4 trajectory from linear regression analyses.

	Unadjusted, β ​(95% CI)​	Fully Adjusted	Model 2
**TSH**	0.001 (-0.004, 0.002)	-0.002 (-0.004, 0.001)	-0.001 (-0.004, 0.001)
**Free T4**	0.069 (0.04, 0.10)**	0.07 (0.04, 0.11)**	0.1 (0.05, 0.10)**
**Free T3**	0.001 (-0.01, 0.01)	0.002 (-0.01, 0.01)	-0.002 (-0.01, 0.01)
**TPO positivity**	-0.005 (-0.012, 0.002)	-0.01 (-0.013, 0.002)	-0.01 (-0.013, 0.001)
**FT3:FT4 ratio**	-0.012 (-0.020, -0.004)*	–	–
**Age**	0.0001 (-0.0002, 0.0003)	0.0001 (-0.0002, 0.0004)	–
**Female**	0.005 (-0.002, 0.011)	0.001 (-0.01, 0.01)	–
**White**	-0.005 (-0.013, 0.003)	-0.004 (-0.012, 0.004)	–
**BMI**	0.0003 (-0.001, 0.001)	0.0001 (-0.001, 0.001)	–
**Smoker**	0.0002 (-0.013, 0.013)	0.001 (-0.01, 0.01)	–
**Walking Index**	-0.001 (-0.002, 0.001)	0.001 (-0.001, 0.003)	–

BMI, body mass index; Smoker, current or within the past 10 years; Walking index is a composite with a higher score meaning better physical function; TPO positivity: the presence of anti-thyroid peroxidase autoantibodies. *P < .05 and **P < .01.

We then estimated the relationship between the same predictor set and phenotype defined by quartiles of change in FT4 (TQ1-TQ4). Thus, TQ1 includes those with the most apparent emerging hypothyroidism patterns, while TQ4 represents the aging stress-response end of the spectrum. The average annual rate of change for FT4 ranged from -1.9% (-0.019 ng/dL/year) in TQ1 to +1.5% (0.013 ng/dl/year) in TQ4. FT4 was significantly lower in TQ1 compared to TQ4 (mean difference 0.94, 95%CI 0.89 – 0.98). Other thyroid variables - FT3, TSH, the annual rate of change in TSH, and TPO-autoantibody status - did not differ between the trajectory quartiles (**Table 1**). Compared to TQ1, TQ4 participants were slightly older, more likely to be female, and less likely to be white, but none of these differences were statistically significant.

**Figure 1:**
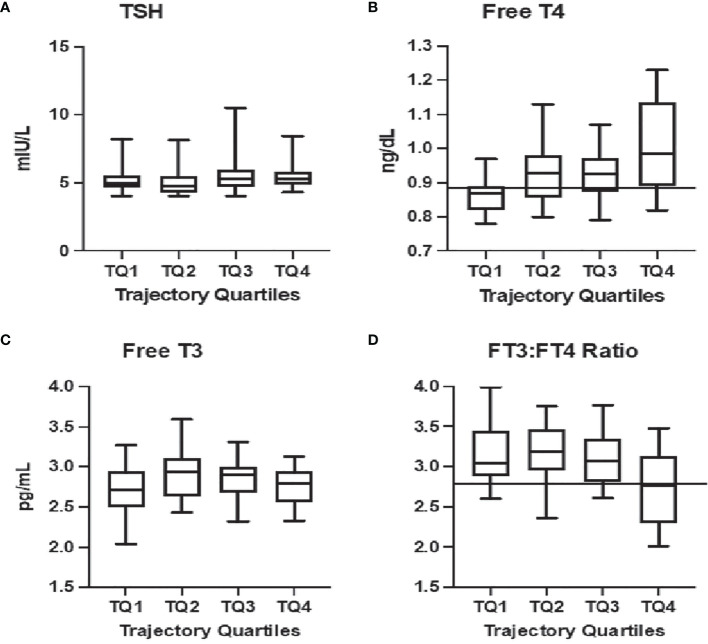
Relationship between current thyroid function tests and HPT axis aging phenotypic: Significant relationships are observed between the trajectory-based phenotype in quartiles from most consistent with emerging hypothyroidism (TQ1) to aging-related stress-responses (TQ4) and free thyroxine [Free T4, **(B)**] as well as the ratio of free triiodothyronine to free thyroxine [FT3:FT4, **(D)**]. Decision tree analysis provided thresholds for predicting membership in TQ1 at a Free T4 = 0.89 ng/dL [solid line, **(B)**] and in TQ4 with a FT3:FT4<2.77 [solid line, **(D)**]. No association is noted with thyrotropin [TSH, **(A)**] or free triiodothyronine [Free T3, **(C)**].

An FT4 value of less than or equal to 0.89 ng/dL (11.45 pmol/L) was the optimal threshold for predicting inclusion in the subclinical hypothyroidism phenotype quartile [AUC=0.75 (0.64, 0.86)] (This coincides with the 24^th^ percentile of the FT4 reference range. No significant thresholds could be defined for TSH (**Figure 1A**) or FT3 (**Figure 1C**). A ratio of FT3:FT4 less than 2.76 predicted inclusion in the aging adaptation phenotype quartile with an [AUC = 0.70 (0.58, 0.83)] The thresholds were found consistent when stratified by TPO antibody status.

## Discussion

Isolated elevated TSH is routinely interpreted in clinical practice as representing subclinical hypothyroidism. Particularly in the setting of symptoms such as fatigue or cold intolerance, older adults are often prescribed thyroid hormone, with temporal trends demonstrating increasing numbers of prescriptions for progressively more mild TSH elevations (8). However, growing evidence that such treatment does not improve symptoms (5) has led to calls for age-specific reference ranges that would redefine subclinical hypothyroidism using the 97.5^th^ percentile of the TSH distribution in older reference populations such as NHANESIII (9, 10). This approach shifts the definition statistically without addressing the underlying heterogeneity in the pathophysiology that can cause TSH elevation, which our longitudinal natural history data has made evident (4), and therefore risks substituting under-inclusion for over-inclusion. Here we have demonstrated that FT4 is better than other additional markers of thyroid status to improve the differentiation of early subclinical hypothyroidism from aging-related stress-response adaptations in those with isolated TSH elevation, since both pathways elevate TSH to a similar degree and tend to defend FT3 in a narrow range. We were intrigued to note that the presence of anti-TPO-antibodies, present in 26% of the study population, did not improve the diagnosis of subclinical hypothyroidism, although the sample size may be limiting our ability to detect a small effect.

The major strength of this study is our ability to phenotype participants based on years of accumulated longitudinal data describing the interacting changes in TSH and FT4. In addition to undertaking multiple repeat measures, the BLSA study design minimizes the risk of misclassification. For example, transient TSH values out of range from acute non-thyroidal illness are highly unlikely, since visits are rescheduled when participants are ill. In addition, diurnal and seasonal variations are minimized for individuals, as visits are scheduled on yearly cycles, and blood is drawn after a supervised overnight fast.

Because the trajectories derived from this data are continuous, phenotypic boundaries cannot be *a priori* delineated. Therefore, we initially analyzed the association between phenotype and current markers of thyroid function on a continuous basis. This confirmed that the trend in FT4 is not a regression to the mean, but a definite physiologic progression such that falling FT4 over time results in a lower FT4, and those with elevated TSH and a trajectory-phenotype most consistent with emerging hypothyroidism have the lowest FT4 values. In order to provide a specific diagnostic definition of subclinical hypothyroidism, we chose quartiles across the phenotype range. Our analyses found that a threshold of FT4<0.89 ng/dL is both sensitive and specific for those in the lowest quartile, with the phenotype suggestive of true early hypothyroidism. This threshold is at the 24^th^ percentile of the reference range for FT4 in our assay. Using equivalencies of mean and standard deviation derived from the manufacturer and published reference ranges, a similar percentile can be determined for other commercially available assays. A preliminary conversion chart for the reported threshold is provided in **Supplemental Table 1** for additional FT4 assays employed in the US and internationally.

Although not as strongly predictive of phenotype, the finding that a smaller FT3:FT4 ratio (less FT3 per unit FT4) is associated with TQ4, supports the physiologic hypothesis that the elevated TSH in those with rising FT4 is a result of HPT axis homeostatic responses to stressors, in which both altered central function and decreased peripheral de-iodination of T4 to T3 are important.

Additional study is needed to demonstrate whether a more targeted definition of subclinical hypothyroidism that uses both elevated TSH and lower FT4 can identify a population which does, in fact, benefit from thyroid hormone replacement. It is not clear that this will be true, since lower FT4 has been associated with both better physical function (11) and better survival (12, 13) in those with euthyroid TSH levels. Research stratifying the treated population by both TSH and FT4 will address whether the presumption of subclinical hypothyroidism among the subgroup of older adults with a TSH above the reference range and FT4 in the lowest quartile of the reference range confers an indication for thyroid hormone supplementation.

## Data Availability Statement

Publicly available datasets were analyzed in this study. This data can be found here: https://www.blsa.nih.gov/.

## Ethics Statement

The studies involving human participants were reviewed and approved by IRB of Intramural Research Program of the National Institutes of Health IRB of Johns Hopkins School of Medicine. The ethics committee waived the requirement of written informed consent for participation.

## Author Contributions

EA, JM, and JSRM had full access to all the data and take full responsibility for the integrity and accuracy of data analyses. EA and JM performed the statistical analyses. LS assisted with the conversion between assays. JSRM, EMS, and JM assisted with the interpretation of the results. JSRM and EMS were involved in the concept, design, and critical revision of the final manuscript, to which LS and JM also contributed. All authors contributed to the article and approved the submitted version.

## Funding

EA, JM, and JSRM: NIA R01AG064256; EMS: NIA Intramural Research Program: JSRM: Turock Family Foundation. Sponsor’s Role: Sponsors did not contribute to the development of the study and paper.

## Conflict of Interest

The authors declare that the research was conducted in the absence of any commercial or financial relationships that could be construed as a potential conflict of interest.

## Publisher’s Note

All claims expressed in this article are solely those of the authors and do not necessarily represent those of their affiliated organizations, or those of the publisher, the editors and the reviewers. Any product that may be evaluated in this article, or claim that may be made by its manufacturer, is not guaranteed or endorsed by the publisher.

## Supplementary Material

Supplementary Table 1 Conversion between assays for threshold FT4 predicting inclusion in TQ1 or TQ4. *Calculated from the manufacturers’ published reference ranges. All conversions use the same factor. ¶ - assuming reference range is central 95^th^ percentile.
