# Supplementary material for: Free Thyroxine Distinguishes Subclinical Hypothyroidism From Other Aging-Related Changes in Those With Isolated Elevated Thyrotropin
Source: Front Endocrinol (Lausanne). 2022 Mar 4;13:858332. doi: 10.3389/fendo.2022.858332 (PMC8931280; doi:10.3389/fendo.2022.858332)
Supplement: Supplementary Table 1 — Conversion between assays for threshold FT4 predicting inclusion in TQ1 or TQ4. *Calculated from the manufacturers’ published reference ranges. All conversions use the same factor. ¶ - assuming reference range is central 95th percentile. [file Table_1.pdf]

Supplemental Table S1: Conversion between assays for threshold FT4 predicting inclusion in TQ1 or TQ4

Laboratory Assays for Free T4 (2.5 – 97.5%) *	Mean (SD)		Threshold TQ1		Threshold TQ4	
	ng/dL	pmol/L	ng/dL	pmol/L	ng/dL	pmol/L
**Abbott Architect i and Alinity i** (0.80 – 1.38 ng/dL) (10.30 – 17.76 pmol/L)	1.09 (0.15)	14.03 (1.93)	0.98	12.61	1.15	14.80
**Beckman Access and UniCel Dxl** (0.61 – 1.12 ng/dL) (7.90 – 14.00 pmol/L)	0.85 (0.12)	10.94 (1.54)	0.76	9.84	0.90	11.60
**Ortho Vitros** (0.90 – 2.08 ng/dL) (11.58 – 26.77 pmol/L)	1.49 (0.30)	19.18 (3.86)	1.28	16.47	1.62	20.85
**Roche Elecsys** (0.93 – 1.70 ng/dL) (11.97 – 21.88 pmol/L)	1.32 (0.20)	16.99 (2.57)	1.18	15.16	1.41	18.09
**Siemens ADVIA Centaur and Atellica IM** (0.89-1.76 ng/dL) (11.46 – 22.65 pmol/L)	1.33 (0.22)	17.12 (2.83)	1.17	15.06	1.42	18.28
**Siemens Vista** (0.72 – 1.26 ng/dL) (9.27 – 16.22 pmol/L)	0.99 (0.14)	12.74 (1.80)	0.89	11.45	1.05	13.51
**Tosoh ST AIA-PACK** (0.75 – 1.54 ng/dL) ¶ (9.65 – 19.82 pmol/L)	1.15 (0.20)	14.80 (2.57)	1.01	12.97	1.24	15.90

*Calculated from the manufacturers' published reference ranges. All conversions use the same factor. ¶ - assuming reference range is central 95th percentile.
